# 611 Comparison of Autologous Skin Cell Suspension to Conventional Surgical Treatment in Burn Patients

**DOI:** 10.1093/jbcr/irae036.244

**Published:** 2024-04-17

**Authors:** booker King, Elisabeth A Carter, Dabbs William, Chris B Agala, Lori Chrisco, Felicia Williams

**Affiliations:** UNC Chapel Hill. NC, Chapel Hill, NC; The University of North Carolina at Chapel Hill, Chapel Hill, NC; UNC Chapel Hill - School of Medicine, Surgery dept, Chapel Hill, NC; NC Jaycee Burn Center, Chapel Hill, NC; UNC Chapel Hill. NC, Chapel Hill, NC; The University of North Carolina at Chapel Hill, Chapel Hill, NC; UNC Chapel Hill - School of Medicine, Surgery dept, Chapel Hill, NC; NC Jaycee Burn Center, Chapel Hill, NC; UNC Chapel Hill. NC, Chapel Hill, NC; The University of North Carolina at Chapel Hill, Chapel Hill, NC; UNC Chapel Hill - School of Medicine, Surgery dept, Chapel Hill, NC; NC Jaycee Burn Center, Chapel Hill, NC; UNC Chapel Hill. NC, Chapel Hill, NC; The University of North Carolina at Chapel Hill, Chapel Hill, NC; UNC Chapel Hill - School of Medicine, Surgery dept, Chapel Hill, NC; NC Jaycee Burn Center, Chapel Hill, NC; UNC Chapel Hill. NC, Chapel Hill, NC; The University of North Carolina at Chapel Hill, Chapel Hill, NC; UNC Chapel Hill - School of Medicine, Surgery dept, Chapel Hill, NC; NC Jaycee Burn Center, Chapel Hill, NC; UNC Chapel Hill. NC, Chapel Hill, NC; The University of North Carolina at Chapel Hill, Chapel Hill, NC; UNC Chapel Hill - School of Medicine, Surgery dept, Chapel Hill, NC; NC Jaycee Burn Center, Chapel Hill, NC

## Abstract

**Introduction:**

In 2018, a device was approved for the treatment of burn injuries. This autologous cell harvesting device is used for the delivery of a regenerative cell suspension, utilizing a patient’s own skin. The study aims to examine outcomes of the device compared to conventional surgical treatment in adult burn patients.

**Methods:**

A retrospective study of burn outcomes in burn patients, admitted from 2015-2022 to a single burn center, was conducted investigating the clinical performance of the autologous skin cell suspension (ASCS) to conventional surgical treatment. Patients ≥18 years of age, with a total body surface area (TBSA) of ≥20% were included. Data were collected utilizing the burn registry and chart review. Patients were divided into two groups: those who received ASCS and those who received conventional surgical treatment (control). We used Fisher’s exact and chi square tests to compare categorical variables, Wilcoxon rank sum test for continuous variables to conduct between-group comparisons, and generalized linear models to assess associations between predictors and outcomes.

**Results:**

A total of 229 patients were identified, 78 were treated with ASCS, and 151 patients received control. Baseline characteristics were comparable in the two groups. The average cost for the ASCS group was $1,244,219 while the control group was $866,072 which was statistically significant. Mean post-hospital plastics clinic visits in the ASCS group was 6.1 and 6.7 in the control group. The mean burn clinic visits was 4.2 among ASCS patients, compared to 3.7 in control group. After adjustment from baseline characteristics, the ASCS group were less likely to be on a ventilator (OR: 0.31, 95% CI: 0.13-0.70); had 1.2 times the number of burn clinic visits than control (IRR: 1.2, 95% CI: 1.03-1.39); LOS for the ASCS group was 0.86 times that of the control group (IRR:0.86, 95% CI: 0.83-0.89); and wound healing was lower (0.82 times) than that of control (IRR:0.82, 95% CI:0.79-0.84); shorter ICU LOS (IRR: 0.56, 95% CI:0.53-0.59); fewer plastics clinic visits (IRR:0.84, 95% CI: 0.70-0.99); fewer vent days (IRR:0.55, 95% CI: 0.52-0.58); 7% higher hospital cost (IRR:1.07, 95% CI: 1.07-1.07). There was no difference in mortality between the groups.

**Conclusions:**

The findings of this study show that receipt of ASCS may be beneficial to some patients by reducing the likelihood of ventilator dependence, quicker wound healing, shorter ICU stay, shorter hospital LOS, fewer plastics clinic visits and slightly higher hospital cost, but no difference in mortality.

There is a need for a multi-center, retrospective study to better understand the benefits of using an autologous skin harvesting device in the treatment of large burns.

**Applicability of Research to Practice:**

Further understanding the impact of surgical interventions will assist in better surgical outcomes in the burn population.

